# Development and internal validation of a LASSO-based nomogram for predicting SAS-defined anxiety symptoms in obstructive sleep apnea

**DOI:** 10.3389/fpsyt.2026.1899687

**Published:** 2026-09-08

**Authors:** Qing Feng, Lingyu Song, Jinqiao Zhang, Jie Zhang, Xiaopei Wang, Rongrong Zhang, Xueyan Wang, Jihua Zhang

**Affiliations:** Department of Otolaryngology, The First Hospital of Hebei Medical University, Shijiazhuang, Hebei, China

**Keywords:** anxiety, longest obstructive apnea duration, nomogram, obstructive sleep apnea, sleep quality

## Abstract

**Background:**

Anxiety symptoms are common but often under-recognized in patients with obstructive sleep apnea (OSA). Conventional polysomnographic severity indices may not fully capture the psychological burden of OSA. This study aimed to develop and internally validate a nomogram for clinically significant anxiety symptoms in patients with OSA.

**Methods:**

A total of 787 patients with OSA were randomly divided into a training cohort (n=551) and a validation cohort (n=236) at a 7:3 ratio. Demographic characteristics, comorbidities, laboratory parameters, patient-reported scales, and polysomnographic variables were collected. Ten-fold cross-validated LASSO logistic regression was used to select predictors from 36 candidate variables. A post-LASSO multivariable logistic model was constructed to develop the nomogram. Discrimination, calibration, and clinical utility were assessed using ROC analysis, bootstrap calibration, Hosmer-Lemeshow testing, and decision curve analysis. Restricted cubic spline analysis examined the association between longest obstructive apnea duration and anxiety symptoms.

**Results:**

Among the 787 included patients, 71 patients (9.0%) had clinically significant anxiety symptoms. Six predictors were retained: gender, cardiovascular disease, hemoglobin, neutrophil-to-lymphocyte ratio, body mass index, and longest obstructive apnea duration. Patients with anxiety symptoms had poorer subjective sleep quality but relatively milder OSA-related parameters, including lower AHI and ODI, shorter longest obstructive apnea duration, and higher minimum SpO_2_. Restricted cubic spline analysis showed a significant overall association between longest obstructive apnea duration and anxiety symptoms (Wald χ²=12.21, df=3, P = 0.0067), with borderline non-linearity (P = 0.0583). Compared with the 60-100-second group, patients with a duration <60 seconds had a higher anxiety risk (OR = 2.84, 95% CI 1.43-5.62, P = 0.0028). The nomogram showed acceptable discrimination, with AUCs of 0.735 and 0.751 in the training and validation cohorts, respectively. Calibration was acceptable, with non-significant Hosmer-Lemeshow tests (P = 0.1056 and 0.4948). Decision curve analysis indicated positive net benefit across broad threshold ranges.

**Conclusion:**

This LASSO-based nomogram showed acceptable performance for predicting SAS-defined clinically significant anxiety symptoms in patients with OSA. Anxiety risk may not be fully captured by conventional respiratory severity indices alone and may reflect a multifactorial risk profile. External validation is warranted.

## 1 Introduction

Obstructive sleep apnea (OSA) is a common sleep-related breathing disorder characterized by recurrent upper airway collapse, intermittent hypoxemia, sleep fragmentation, and repeated arousals during sleep. Recent studies have shown that OSA imposes a substantial burden in both the general population and hospitalized patients and is closely associated with increased healthcare utilization (1–4). OSA has been linked to a wide range of adverse outcomes, including cardiovascular disease, metabolic dysfunction, neurocognitive impairment, reduced quality of life, and increased mortality risk (5–10). Although the clinical evaluation of OSA has traditionally relied on respiratory indices such as the apnea-hypopnea index, oxygen desaturation index, and nocturnal oxygen saturation, accumulating evidence suggests that the burden of OSA extends beyond respiratory pathophysiology and includes important mental health consequences (11–14).

Anxiety symptoms are frequently observed in patients with OSA and may worsen insomnia complaints, impair daytime functioning, reduce adherence to positive airway pressure therapy, and adversely influence long-term clinical outcomes (15–18). However, the relationship between OSA severity and anxiety remains incompletely understood. Some studies have suggested that anxiety symptoms may be associated with nocturnal hypoxemia, autonomic activation, inflammatory responses, or sleep fragmentation, whereas others have reported weak or inconsistent associations between anxiety and conventional polysomnographic severity indices (19–22). These inconsistent findings suggest that anxiety symptoms in patients with OSA may not be fully explained by AHI or oxygen desaturation burden alone. Instead, anxiety in OSA may reflect multidimensional clinical characteristics involving sex-related susceptibility, cardiovascular comorbidities, obesity, systemic inflammation, hematological alterations, and specific respiratory-event characteristics (27–31). Nevertheless, clinically applicable tools for individualized anxiety risk stratification in patients with OSA remain limited. In current clinical practice, anxiety symptoms in patients with OSA are generally identified using self-reported psychological questionnaires or clinical interviews, particularly when patients present with emotional distress, insomnia, or other subjective complaints. However, psychological screening is not always systematically incorporated into routine sleep-medicine or otolaryngology assessment, which often prioritizes PSG-derived respiratory severity indices such as AHI, ODI, and oxygen saturation. Consequently, patients with clinically significant anxiety symptoms may be overlooked, especially when conventional respiratory indices suggest only mild or moderate disease severity. A prediction model based on routinely available clinical information may therefore help identify patients who warrant further psychological screening.

To address this clinical need, the primary aim of the present study was to develop and internally validate a clinically applicable nomogram for predicting clinically significant anxiety symptoms in patients with OSA. We used ten-fold cross-validated LASSO logistic regression to select candidate predictors from demographic characteristics, comorbidities, laboratory parameters, and OSA-related variables, and subsequently constructed a post-LASSO multivariable logistic regression model. The discrimination, calibration, and clinical utility of the nomogram were evaluated in both the training and validation cohorts. Separately, we explored the association between longest obstructive apnea duration and anxiety symptoms to assess whether specific respiratory-event characteristics might provide information beyond conventional respiratory severity indices. This exploratory analysis was intended to generate hypotheses rather than to establish a causal biological mechanism.

## 2 Materials and methods

### 2.1 Study design and study population

This was a single-center, retrospective observational study. The source population consisted of consecutive adult patients hospitalized in the Department of Otolaryngology at the First Hospital of Hebei Medical University between January 2025 and December 2025 for evaluation of snoring, suspected sleep-disordered breathing, OSA-related symptoms, or upper-airway obstruction. During hospitalization, overnight polysomnography was routinely performed as part of the clinical evaluation. Clinical data were retrospectively retrieved from the hospital electronic medical record (EMR) system and laboratory information system (LIS). No additional interventions or examinations were performed for the purpose of this study.

The inclusion criteria were as follows: ① age ≥18 years; ② completion of overnight polysomnography (PSG) and diagnosis of OSA based on an AHI of ≥5 events/h; ③ completion of patient-reported outcome assessments at the time of PSG or during the same clinical encounter, including the Zung Self-Rating Anxiety Scale (SAS), Epworth Sleepiness Scale (ESS), and Pittsburgh Sleep Quality Index (PSQI); and ④ availability of complete clinical data, laboratory parameters, and PSG reports for subsequent model development.

The exclusion criteria were as follows: ① a previous diagnosis of severe mental disorders, such as schizophrenia or bipolar disorder, or current treatment with anxiolytics, antidepressants, or other psychotropic medications; ② previous treatment for OSA, including continuous positive airway pressure (CPAP) therapy, upper airway surgery, or other disease-specific interventions; ③ severe hepatic, renal, cardiac, or pulmonary failure, malignant tumors, acute infection, or other conditions that could substantially affect affective status, sleep architecture, or laboratory test results; and ④ missing key variables or incomplete records, including unavailable major PSG parameters, missing anxiety scale scores, or unavailable essential laboratory parameters.

**Figure 1:**
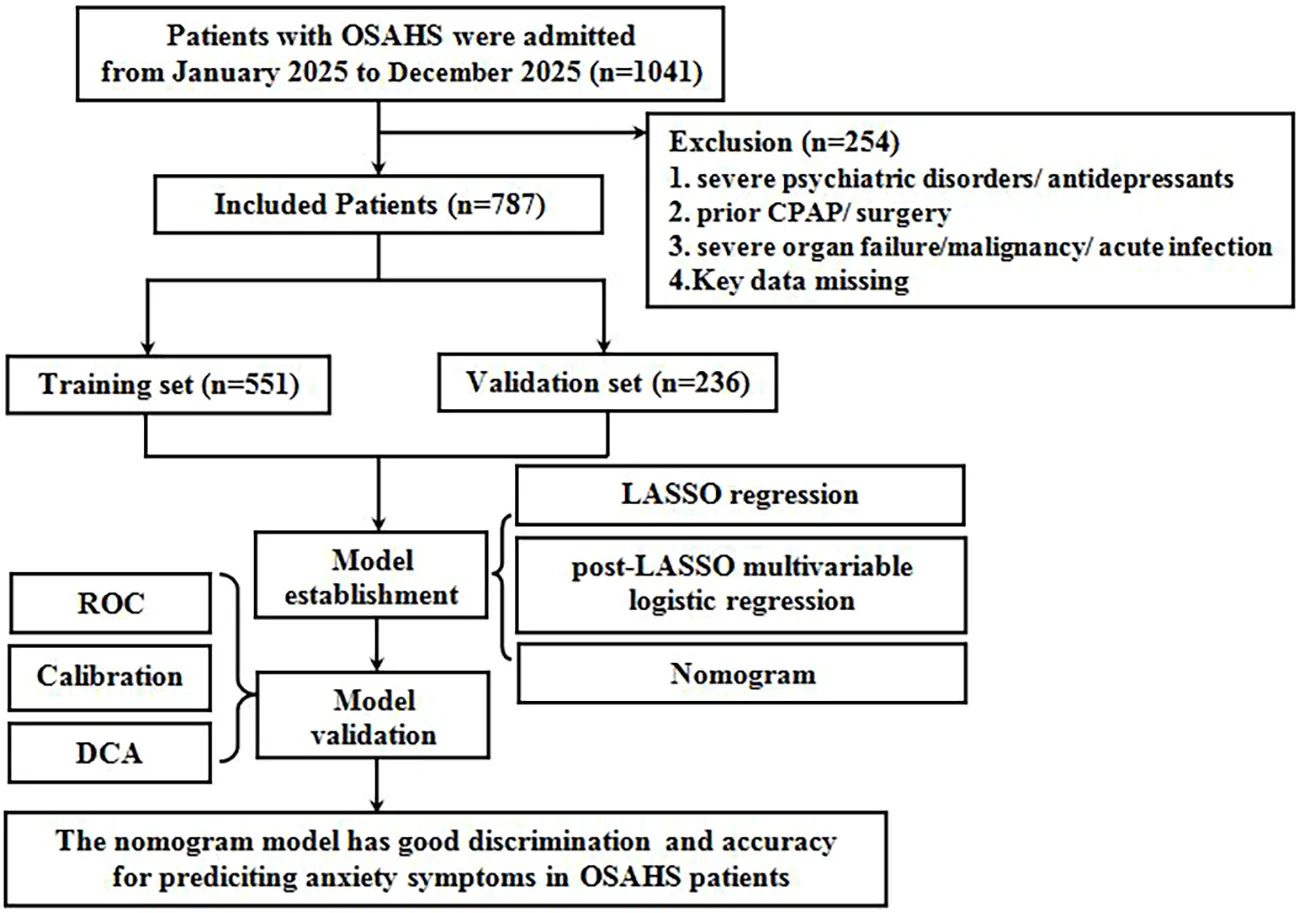
Flowchart of participant selection, data extraction, model development, and validation.

The study protocol was reviewed and approved by the Ethics Committee of the First Hospital of Hebei Medical University (*Approval No. (2025): Yanshen No. (270)*). Because this was a retrospective analysis of de-identified data, the requirement for written informed consent was waived by the ethics committee. The study was conducted in accordance with the principles of the Declaration of Helsinki. The study flowchart is presented in **Figure 1**.

### 2.2 Data collection and clinical evaluation

All data were independently extracted from the EMR and LIS systems by two investigators who had received standardized training. After data extraction, the two investigators cross-checked all data entries. Any discrepancies or uncertainties were resolved by a third investigator after reviewing the original source records. To maintain consistency in data sources, only examinations and assessments completed as part of routine clinical care were included in this study. No additional specimens, tests, or assessments were obtained for research purposes.

#### 2.2.1 Demographic characteristics and clinical history

Data on demographic characteristics, lifestyle factors, and medical history were collected. Demographic and clinical variables included age, gender, disease duration, body mass index (BMI), smoking history, history of alcohol consumption, and memory impairment. Medical history and comorbidities included hypertension, diabetes mellitus, cardiovascular disease, inferior turbinate hypertrophy, fatty liver disease, abnormal pulmonary imaging findings, chronic rhinitis, and hyperlipidemia. These comorbidities were identified based on previous medical records, discharge diagnoses, imaging findings, laboratory results, or specialist-confirmed diagnoses.

#### 2.2.2 Polysomnographic assessment

PSG data were obtained from overnight PSG reports performed at the time of the initial diagnosis of OSA. All PSG examinations were conducted in a standardized sleep laboratory. The recorded signals included electroencephalography, electrooculography, chin electromyography, electrocardiography, oronasal airflow, thoracoabdominal movements, oxygen saturation, body position, snoring, and other relevant parameters.

Respiratory events during sleep were manually scored by experienced sleep technicians according to the American Academy of Sleep Medicine (AASM) Scoring Manual, Version 3.0. Obstructive apnea was defined as a ≥90% reduction in oronasal airflow from baseline lasting for at least 10 seconds. Hypopnea was defined as a ≥30% reduction in airflow lasting for at least 10 seconds, accompanied by either a ≥3% decrease in oxygen saturation or an arousal.

The PSG parameters extracted in this study included the apnea-hypopnea index (AHI), longest obstructive apnea duration, oxygen desaturation index (ODI), and lowest oxygen saturation (min SpO_2_). AHI was used to indicate the average number of apnea and hypopnea events per hour of sleep. ODI was used to assess nocturnal oxygen desaturation burden. Min SpO_2_ represented the lowest oxygen saturation recorded during overnight monitoring. Longest obstructive apnea duration was defined as the maximum duration of a single obstructive apnea event recorded during overnight PSG and was expressed in seconds.

#### 2.2.3 Patient-reported outcome assessments

Patient-reported outcome data were extracted from routine clinical assessment records completed at admission, at the time of PSG, or during the same clinical encounter. Daytime sleepiness was assessed using the Epworth Sleepiness Scale (ESS). Subjective sleep quality was evaluated using the Pittsburgh Sleep Quality Index (PSQI), with higher PSQI scores indicating poorer sleep quality.

#### 2.2.4 Laboratory measurements

Laboratory data were retrieved from the LIS system. When multiple measurements were available, the results obtained closest to the PSG examination were used. In general, laboratory parameters were measured within 24 hours before or after PSG, preferably using fasting morning venous blood samples.

The extracted metabolic and biochemical parameters included high-density lipoprotein cholesterol (HDL-C), fasting blood glucose (FBG), triglycerides (TG), low-density lipoprotein cholesterol (LDL-C), total cholesterol (TC), urinary creatinine, and uric acid (UA). Thyroid function parameters included free thyroxine (FT4), free triiodothyronine (FT3), and thyroid-stimulating hormone (TSH). Hematological parameters included hemoglobin (Hb), platelet count, and white blood cell count (WBC).

In addition, the neutrophil-to-lymphocyte ratio (NLR) was calculated from complete blood count results using the following formula:


NLR=neutrophil count/lymphocyte count

Estimated glomerular filtration rate (eGFR) was calculated using the CKD-EPI equation. All laboratory tests were performed in the institutional clinical laboratory according to standard operating procedures.

### 2.3 Outcome definition

Anxiety symptoms were assessed using the Zung Self-Rating Anxiety Scale (SAS), a self-report screening instrument for anxiety symptoms. Patients were classified as having elevated anxiety symptoms according to the established cutoff for the SAS standard score (SAS standardized score ≥50). Higher SAS scores indicated more severe anxiety symptoms. Because the SAS is a screening scale rather than a structured psychiatric diagnostic interview, the primary outcome of this study should be interpreted as SAS-defined clinically significant anxiety symptoms rather than a formal diagnosis of anxiety disorder. The SAS was used because it was routinely administered as part of the clinical psychological assessment for patients with OSA in our center during the study period. Other commonly used anxiety screening tools, such as the Generalized Anxiety Disorder-7 scale (GAD-7), were not systematically collected in this retrospective dataset and therefore could not be analyzed.

### 2.4 Statistical analysis and model development

The development, validation, and reporting of the prediction model were guided by the TRIPOD recommendations. A completed TRIPOD reporting checklist is provided in **Supplementary Table S6**. All statistical analyses were performed using R software (version 4.3.1). The study cohort was randomly divided into a training cohort and a validation cohort at a ratio of 7:3. This split-sample approach was used to provide an internal validation subset that was not involved in model fitting.

Before model development, missing data were assessed for all candidate predictors. The number and proportion of missing values for each candidate predictor in the training and validation cohorts are summarized in **Supplementary Table S4**. Complete-case analysis was not used. For continuous variables, missing values were imputed using the median value derived from the training cohort, whereas categorical variables were imputed using the mode value derived from the training cohort. The same training-cohort-derived imputation values were applied to the validation cohort to avoid information leakage. No outcome values were imputed.

Baseline characteristics were summarized using the tableone package. Normally distributed continuous variables were presented as mean ± standard deviation (mean ± SD), whereas non-normally distributed variables were presented as median and interquartile range [M (P25, P75)]. Categorical variables were reported as counts and percentages, n (%). Between-group comparisons were performed using Student’s t-test or the Mann–Whitney U test for continuous variables, as appropriate, and the χ² test or Fisher’s exact test for categorical variables.

LASSO logistic regression was performed using the glmnet package in R to select predictors for SAS-defined clinically significant anxiety symptoms. The outcome variable was coded as 1 for patients with SAS-defined clinically significant anxiety symptoms and 0 for those without. A total of 36 candidate predictors were entered into the LASSO model. Binary categorical variables were coded as 0/1, and continuous variables were entered as numeric variables. During LASSO fitting, predictors were standardized to place variables on a comparable scale. Ten-fold cross-validation was used to determine the optimal penalty parameter λ. The λ_min criterion, defined as the value of λ that minimized the cross-validated binomial deviance, was selected because the primary objective was prediction performance. Predictors with non-zero coefficients at λ_min were retained and subsequently included in the post-LASSO multivariable logistic regression model. No additional stepwise selection based on individual P values was performed after LASSO selection.

A nomogram was constructed based on the coefficients of the post-LASSO multivariable logistic regression model and generated using the rms package. Model discrimination was assessed using the area under the receiver operating characteristic curve (AUC) and the concordance index (C-index). Calibration was evaluated using calibration curves and the Hosmer–Lemeshow goodness-of-fit test. Clinical utility was assessed using decision curve analysis (DCA). Given the modest number of anxiety events and the potential statistical inefficiency of split-sample validation, bootstrap-based internal validation using the entire analytical cohort was additionally performed with 1,000 resamples to estimate optimism-corrected model performance, including the AUC/C-index, calibration intercept, calibration slope, and Brier score. All statistical tests were two-sided, and P < 0.05 was considered statistically significant for descriptive comparisons.

## 3 Results

### 3.1 Baseline characteristics of the study population

A total of 787 patients with OSA were included in this study. The mean age of the study population was 40.0 years, and 89.3% of the patients were male. Based on anxiety scale scores, patients were classified into an anxiety-symptom group and a non-anxiety-symptom group. Gender and drinking history differed significantly between the two groups (P < 0.05), with the anxiety-symptom group showing higher proportions of female patients and non-drinkers. Compared with the non-anxiety-symptom group, the anxiety-symptom group had a higher prevalence of cardiovascular disease but a lower prevalence of hyperlipidemia. In addition, OSA-related parameters indicated relatively less severe disease in the anxiety-symptom group, including lower AHI and ODI, shorter longest obstructive apnea duration, and higher minimum SpO_2_. However, patients in the anxiety-symptom group had poorer subjective sleep quality, as reflected by higher PSQI scores. Significant between-group differences were also observed in several laboratory parameters, including FT3, TC, LDL-C, and Hb (P < 0.05), as shown in **Table 1**.

**Table 1:** Demographics and baseline data.

Variables	Total	Non-anxiety	Anxiety	P value	t/(Z)/[χ²] value
N	787	716	71		
Demographic and clinical characteristics:					
Disease duration (year)	10.00 (4.00, 10.00)	10.00(4.75, 10.00)	8.00 (2.00, 10.00)	0.156	(-1.417)
Age (year)	40.00 (36.00, 46.00)	40.00 (36.00, 45.00)	41.00 (34.50, 48.00)	0.825	(-0.220)
Gender/n (%)					
Female	84 (10.7)	60 (8.4)	24 (33.8)	<0.001	[43.789]
Male	703 (89.3)	656 (91.6)	47 (66.2)		
Smoking history/n (%)					
No	517 (65.7)	464 (64.8)	53 (74.6)	0.125	[2.777]
Yes	270 (34.3)	252 (35.2)	18 (25.4)		
Alcohol consumption history/n (%)					
No	474 (60.2)	419 (58.5)	55 (77.5)	0.003	[9.679]
Yes	313 (39.8)	297 (41.5)	16 (22.5)		
BMI (kg/m^2^)	28.37 (25.79, 31.14)	28.37 (25.95, 31.14)	28.34 (25.27, 31.60)	0.931	(-0.086)
Hypertension/n (%)					
No	518 (65.8)	470 (65.6)	48 (67.6)	0.840	[0.111]
Yes	269 (34.2)	246 (34.4)	23 (32.4)		
DM/n (%)					
No	729 (92.6)	666 (93.0)	63 (88.7)	0.280	[1.737]
Yes	58 (7.4)	50 (7.0)	8 (11.3)		
Cardiovascular disease/n (%)					
No	677 (86.0)	622 (86.9)	55 (77.5)	0.045	[4.754]
Yes	110 (14.0)	94 (13.1)	16 (22.5)		
Inferior turbinate hypertrophy/n (%)					
No	44 (5.6)	36 (5.0)	8 (11.3)	0.056	[4.765]
Yes	743 (94.4)	680 (95.0)	63 (88.7)		
Fatty liver disease/n (%)					
No	518 (65.8)	468 (65.4)	50 (70.4)	0.468	[0.735]
Yes	269 (34.2)	248 (34.6)	21 (29.6)		
Abnormal pulmonary imaging findings/n (%)					
No	100 (12.7)	91 (12.7)	9 (12.7)	1.000	[0.000]
Yes	687 (87.3)	625 (87.3)	62 (87.3)		
Chronic rhinitis/n (%)					
No	645 (82.0)	587 (82.0)	58 (81.7)	1.000	[0.004]
Yes	142 (18.0)	129 (18.0)	13 (18.3)		
Hyperlipidemia/n (%)					
No	514 (65.3)	459 (64.1)	55 (77.5)	0.034	[5.088]
Yes	273 (34.7)	257 (35.9)	16 (22.5)		
Memory impairment/n (%)					
No	163 (20.7)	151 (21.1)	12 (16.9)	0.498	[0.690]
Yes	624 (79.3)	565 (78.9)	59 (83.1)		
OSA-related parameters:					
AHI (events/h)	57.90 (41.10, 69.60)	57.90 (43.40, 70.32)	43.10 (24.35, 65.15)	0.002	(-3.162)
Longest Obstructive Apnea Duration (s)	72.60 (61.60, 85.75)	72.60 (62.38, 86.12)	72.00 (55.10, 76.90)	0.011	(-2.533)
ODI (events/h)	54.40 (39.40, 65.50)	54.40 (41.82, 67.05)	54.40 (23.05, 56.60)	0.008	(-2.667)
minSpO_2_ (%)	67.00 (55.00, 78.00)	67.00 (54.00, 77.00)	75.00 (62.50, 83.00)	0.001	(-3.251)
Patient-reported outcomes:					
PSQI	8.00 (6.00, 10.00)	8.00(6.00, 10.00)	8.00 (8.00, 12.00)	<0.001	(-3.542)
ESS	7.00(4.00, 11.00)	7.00 (4.00, 11.00)	7.00 (6.50, 9.50)	0.836	(-0.206)
Laboratory parameters					
Urinary Creatinine (mmol/L)	17.70 (8.80, 26.50)	17.70 (8.80, 26.50)	17.70 (8.80, 26.50)	0.528	(-0.631)
HDL-C (mmol/L)	1.13 (0.97, 1.30)	1.13 (0.97, 1.29)	1.12 (1.00, 1.35)	0.921	(-0.099)
FBG (mmol/L)	5.15 (4.77, 5.64)	5.16 (4.78, 5.66)	5.11 (4.71, 5.54)	0.336	(-0.962)
TG (mmol/L)	1.94 (1.33, 2.88)	1.94 (1.34, 2.90)	1.84 (1.06, 2.54)	0.119	(-1.559)
FT4 (pmol/L)	11.15 (10.22, 12.17)	11.16 (10.20, 12.13)	10.94 (10.27, 12.45)	0.917	(-0.104)
FT3 (pmol/L)	5.54 (5.13, 5.99)	5.57 (5.14, 6.01)	5.33 (4.86, 5.66)	0.004	(-2.880)
TC (mmol/L)	5.05 (4.46, 5.85)	5.07 (4.51, 5.86)	4.65 (3.91, 5.71)	0.013	(-2.489)
UA (μmol/L)	402.40 ± 92.97	404.20 ± 92.07	384.23 ± 100.52	0.084	1.729
TSH (mIU/L)	1.72 (1.25, 2.40)	1.71 (1.24, 2.37)	1.79 (1.43, 2.48)	0.183	(-1.332)
LDL-C (mmol/L)	3.09 (2.64, 3.58)	3.11 (2.67, 3.60)	2.91 (2.41, 3.46)	0.021	(-2.309)
NLR	1.88 (1.49, 2.48)	1.86 (1.50, 2.46)	1.94 (1.42, 2.62)	0.292	(-1.054)
Hb (g/L)	155.00 (146.00, 163.00)	155.00 (147.00, 164.00)	145.00 (134.00, 157.00)	<0.001	(-5.263)
Platelet Count (×10^9^/L)	234.00 (202.00, 274.00)	235.00 (203.00, 274.00)	222.00 (199.50, 271.50)	0.332	(-0.971)
WBC (×10^9^/L)	6.50 (5.45, 7.70)	6.55 (5.50, 7.70)	6.50 (5.30, 7.75)	0.691	(-0.397)
eGFR (mL/min/1.73m²)	116.14 (107.40, 123.84)	116.09 (107.47, 123.47)	118.04 (106.02, 130.83)	0.168	(-1.379)

Data are presented as mean ± standard deviation, median (interquartile range), or number (percentage), as appropriate. P values were calculated using Student’s t test or Mann-Whitney U test for continuous variables and χ² test or Fisher’s exact test for categorical variables.

AHI, Apnea-Hypopnea Index; ODI, Oxygen Desaturation Index; PSQI, Pittsburgh Sleep Quality Index; ESS, Epworth Sleepiness Scale; HDL-C, high-density lipoprotein cholesterol; LDL-C, low-density lipoprotein cholesterol; eGFR, estimated glomerular filtration rate; NLR, neutrophil-to-lymphocyte ratio; WBC, white blood cell count.

Using R software, the study population was randomly divided into a training cohort and a validation cohort at a ratio of 7:3. The training cohort included 551 patients, and the validation cohort included 236 patients. Baseline characteristics were compared between the training and validation cohorts to assess the comparability of the two datasets after random splitting. No statistically significant differences were observed between the two cohorts in baseline characteristics (P > 0.05), as shown in **Supplementary Table S1**.

### 3.2 Selection of predictors for anxiety symptoms in patients with OSA using LASSO regression

**Figure 2:**
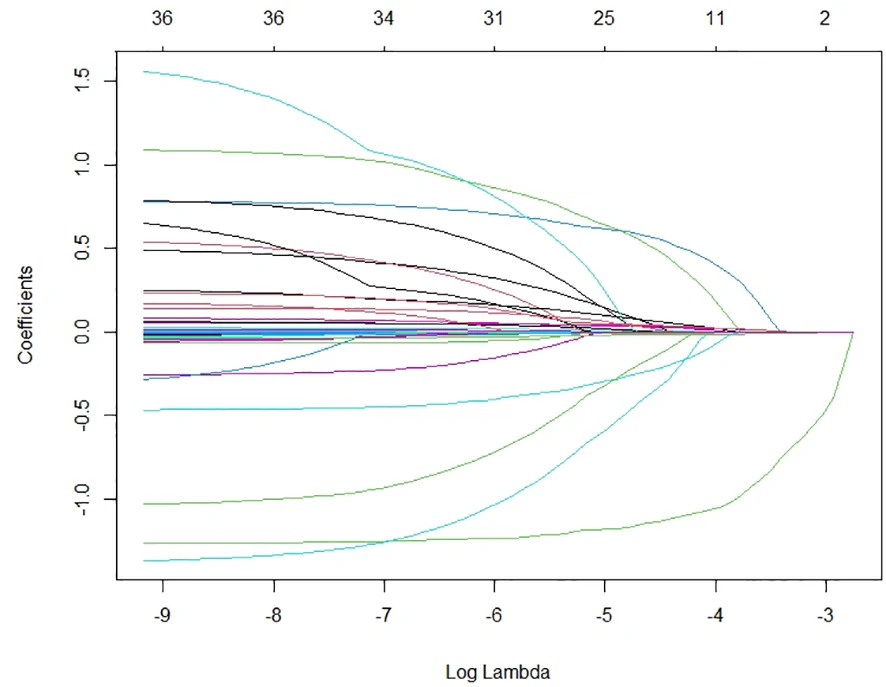
Coefficient profile plot for predictors in the LASSO logistic regression model. Coefficient trajectories are shown as a function of the log-transformed penalty parameter (log λ). As λ increases, coefficients are progressively shrunk toward zero. Predictors with non-zero coefficients at λ_min were retained for post-LASSO model development.

**Figure 3:**
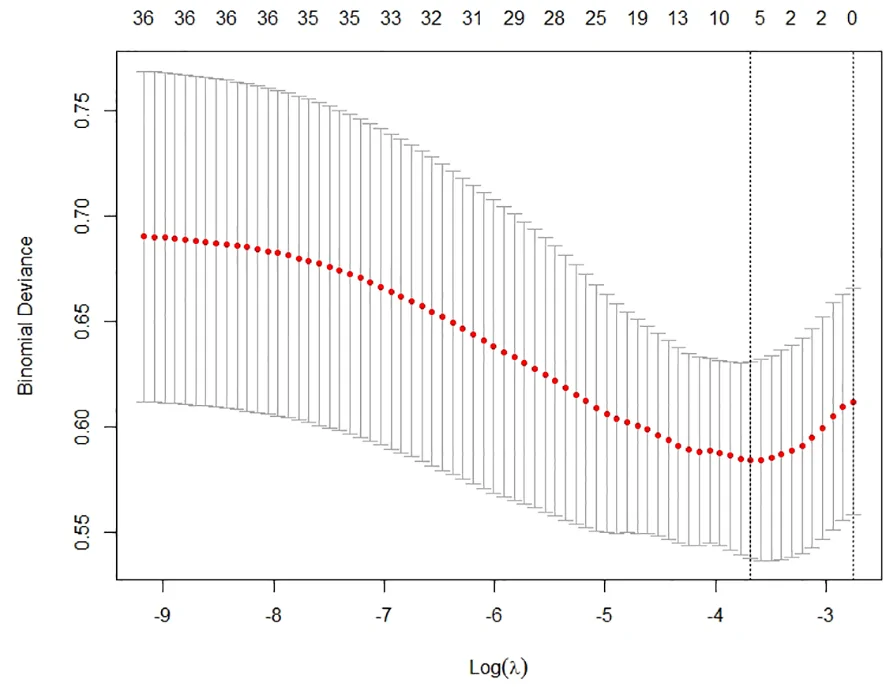
Ten-fold cross-validation plot for selecting the optimal penalty parameter (λ) in the LASSO model. The plot shows the mean cross-validated binomial deviance ( ± 1 standard error) across a grid of λ values. The vertical reference line indicates λ_min, which was selected to optimize predictive performance; predictors with non-zero coefficients at λ_min were carried forward to the post-LASSO multivariable logistic regression model.

In the training cohort, LASSO regression was applied to the included variables to identify candidate predictors associated with anxiety symptoms. The LASSO coefficient profile plot and cross-validation plot are shown in **Figures 2**, **3**, respectively. Of the 36 candidate predictors entered into the LASSO logistic regression, six predictors had non-zero coefficients at the λ_min criterion and were retained for post-LASSO model construction: gender, cardiovascular disease, hemoglobin, neutrophil-to-lymphocyte ratio, BMI, and longest obstructive apnea duration.

### 3.3 Post-LASSO multivariable model and nomogram development

**Figure 4:**
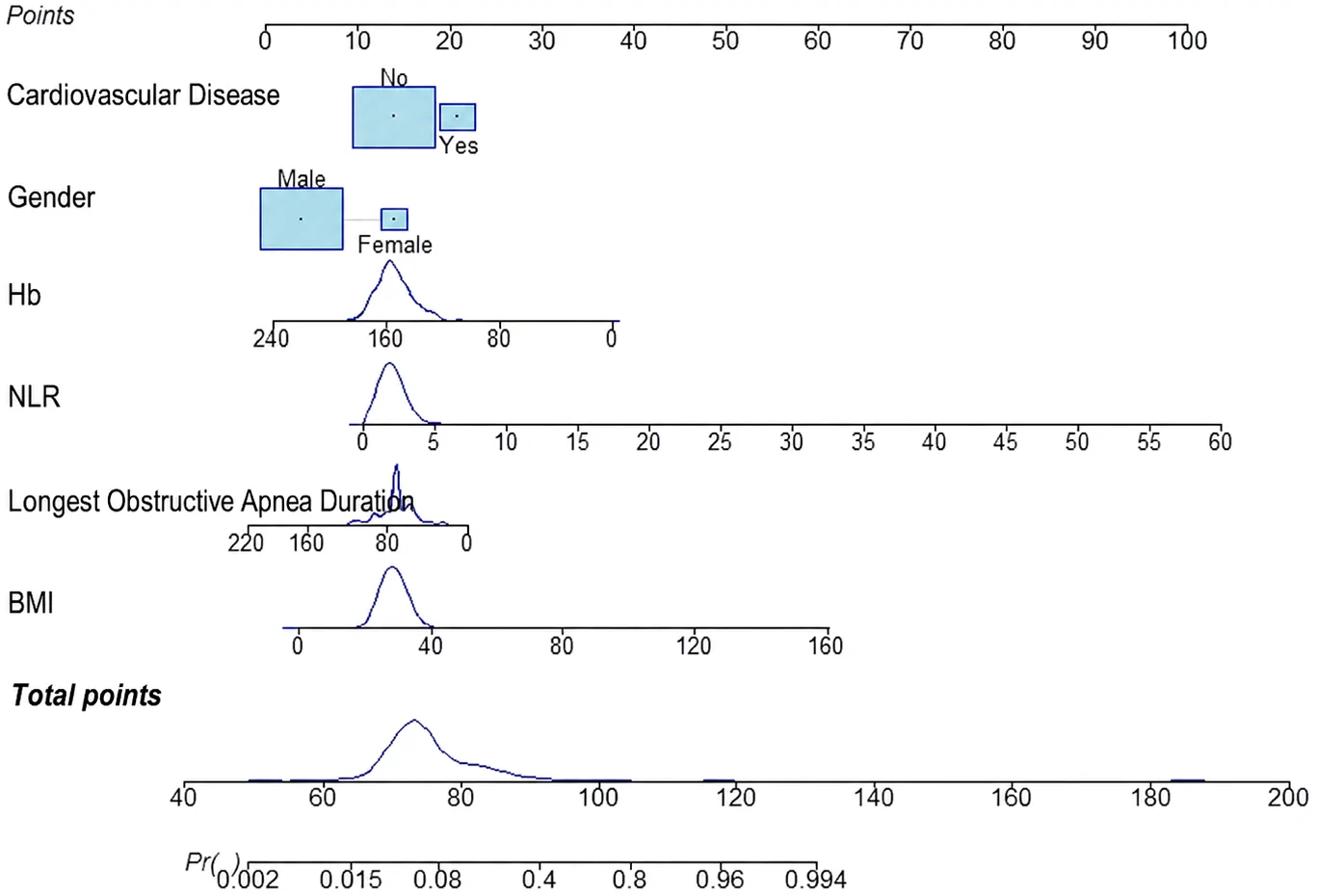
Nomogram for predicting anxiety symptoms in OSA.

All six predictors retained by LASSO selection were entered into the post-LASSO multivariable logistic regression model to estimate regression coefficients, odds ratios (ORs), and predicted probabilities (**Table 2**). Predictor retention was determined by the LASSO selection process at λ_min rather than by statistical significance testing in the multivariable model. Because the primary objective of this model was prediction rather than etiological inference, all predictors retained by LASSO selection were included in the post-LASSO multivariable logistic regression model irrespective of their individual P values. Accordingly, the statistical significance of individual predictors should not be interpreted as the sole criterion for their contribution to risk prediction. Based on the fitted multivariable model, a nomogram was developed to estimate the probability of clinically significant anxiety symptoms in individual patients (**Figure 4**). In the nomogram, each predictor corresponds to a specific point value on the top scale. The total score was calculated by summing the points assigned to all predictors. This total score corresponded to the predicted probability scale at the bottom of the figure, ranging from 0.0 to 1.0, which represented the estimated risk of anxiety. Higher total scores indicated a greater probability of anxiety symptoms (**Figure 4**).

**Table 2:** Post-LASSO multivariable logistic regression model for clinically significant anxiety symptoms in the training cohort.

Subjects	B value	SE value	Wald χ2 values	P value	OR value	95% CI
Longest Obstructive Apnea Duration	-0.013	0.008	2.473	0.116	0.987	0.972-1.003
Cardiovascular Disease	1.085	0.404	7.202	0.007	2.959	1.340-6.535
Gender	-1.196	0.525	5.189	0.023	0.303	0.108-0.846
Hb	-0.028	0.014	3.859	0.049	0.972	0.946-1.000
NLR	0.335	0.178	3.556	0.059	1.399	0.987-1.982
BMI	-0.004	0.044	0.008	0.928	0.996	0.913-1.086
Constant	3.067	2.207	1.931	0.165	21.474	

All predictors in this table were pre-selected by LASSO at λ_min based on predictive informativeness and were entered into the multivariable logistic regression model regardless of statistical significance to avoid post-selection bias. Coefficients and ORs are reported to facilitate interpretation and nomogram construction. OR, odds ratio; CI, confidence interval; SE, standard error.

### 3.4 Non-linear association between longest obstructive apnea duration and anxiety symptoms

To further examine the relationship between longest obstructive apnea duration and clinically significant anxiety symptoms, we performed restricted cubic spline (RCS) analysis. The overall association was statistically significant (Wald χ² = 12.21, df = 3, P = 0.0067), whereas the non-linear component showed only a borderline trend toward non-linearity (P for non-linearity = 0.0583). The estimated risk was lowest at approximately 91.73 seconds and then increased slightly as the duration became longer, suggesting a possible asymmetric U-shaped pattern; however, this pattern should be interpreted cautiously (**Supplementary Figure S1**). In a supplementary categorical analysis, longest obstructive apnea duration was categorized into three groups: <60 seconds, 60–100 seconds, and >100 seconds. With the 60–100 seconds group as the reference, the <60 seconds group had a significantly higher risk of anxiety symptoms (OR = 2.84, 95% CI 1.43-5.62, P = 0.0028). In contrast, the >100 seconds group showed a numerically higher but non-significant risk (OR = 1.26, 95% CI 0.46-3.50, P = 0.654) (**Supplementary Table S2**). These findings suggest that a shorter longest obstructive apnea duration may be associated with a higher risk of anxiety symptoms, whereas the observed non-linear pattern should be considered exploratory. Comparison of the linear and restricted cubic spline models is provided in **Supplementary Table S3**.

### 3.5 Evaluation and validation of the nomogram in the training and validation cohorts

**Figure 5:**
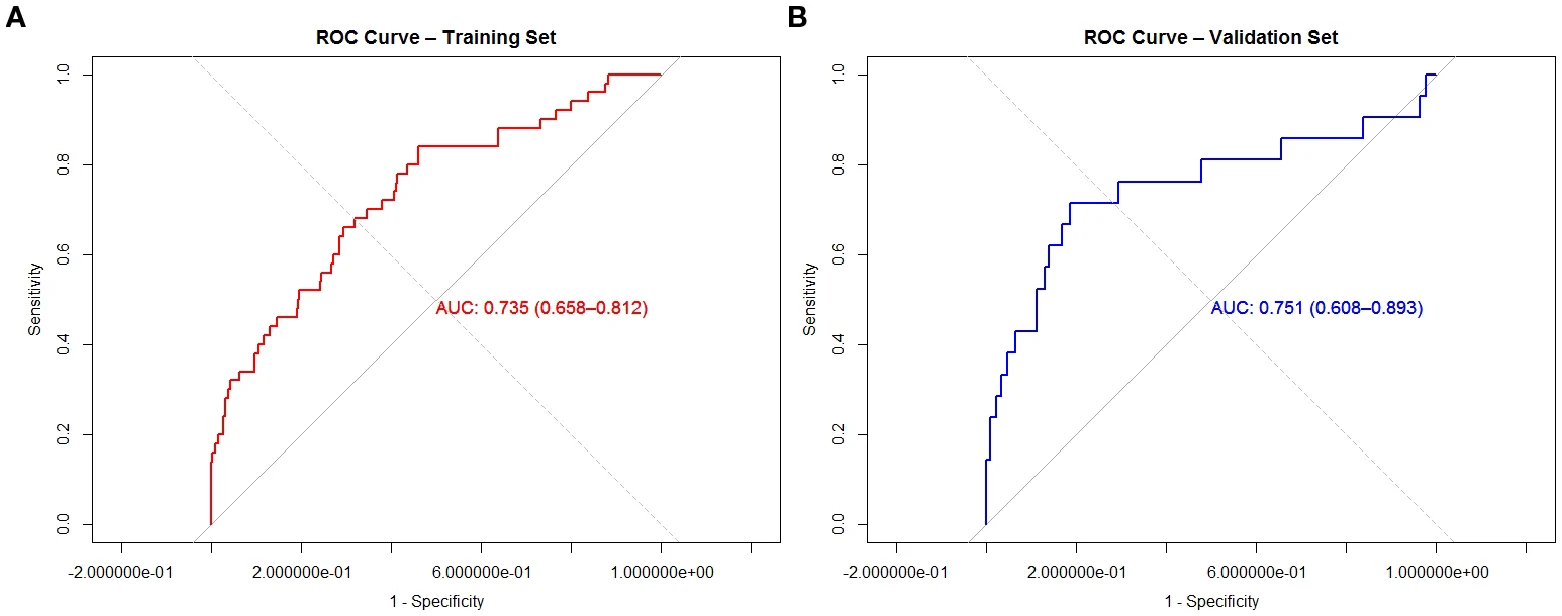
ROC curves for predicting anxiety symptoms among patients with OSA in the training cohort **(A)** and validation cohort **(B)**.

The discriminative ability of the nomogram was evaluated by receiver operating characteristic (ROC) curve analysis and the area under the curve (AUC). The AUC was 0.735 (95% CI: 0.658-0.812) in the training cohort and 0.751 (95% CI: 0.608-0.893) in the validation cohort. These findings indicated that the nomogram had acceptable discrimination for predicting anxiety symptoms in patients with OSA (**Figure 5**).

**Figure 6:**
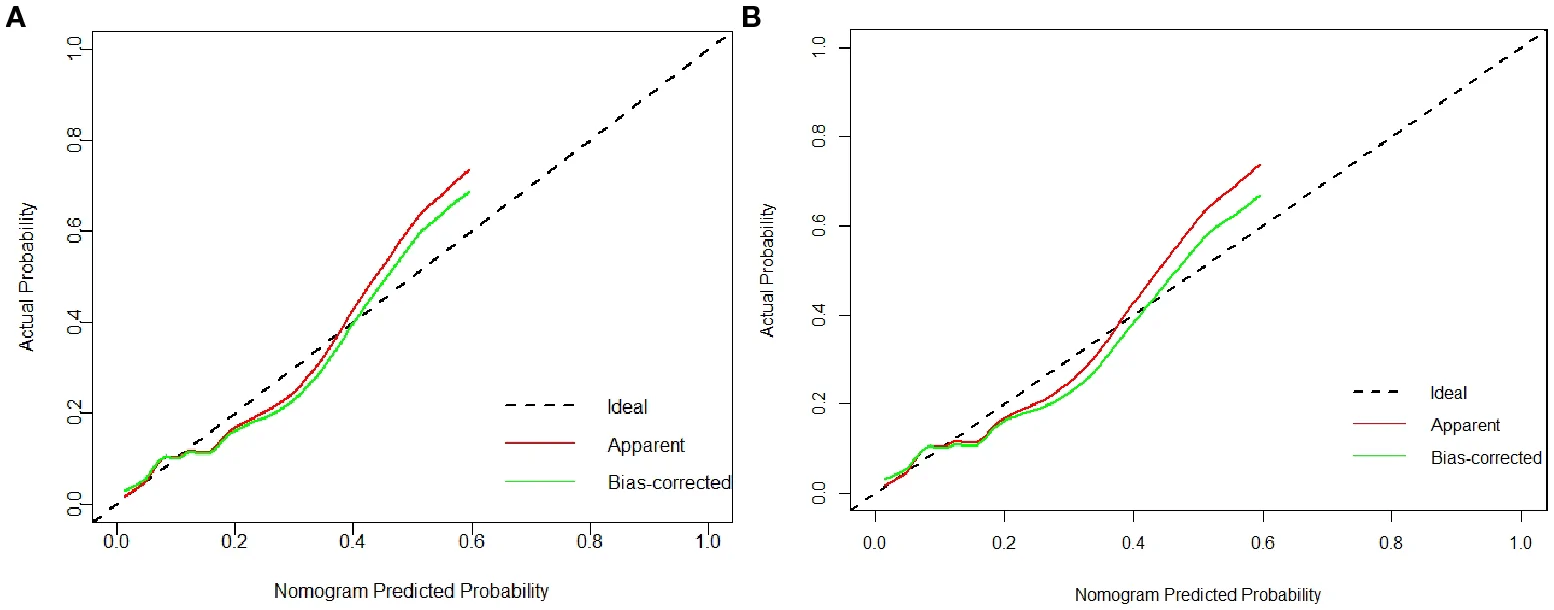
Calibration curves of the nomogram prediction in the training cohort **(A)** and validation cohort **(B)**.

Calibration of the model was assessed using bootstrap resampling with 1,000 repetitions. The calibration curves (**Figure 6**) showed that, in both the training and validation cohorts, the bias-corrected curve (green) and apparent curve (red) showed good agreement with the ideal line (black), indicating consistency between predicted and observed probabilities and suggesting stable model performance. In addition, the Hosmer-Lemeshow goodness-of-fit test showed no statistically significant differences between predicted and observed probabilities in either the training cohort or the validation cohort, with P values of 0.1056 and 0.4948, respectively. These results further supported the acceptable calibration of the model for estimating the risk of anxiety symptoms. In addition to the split-sample validation, bootstrap-based internal validation using the entire analytical cohort with 1,000 resamples was performed to estimate optimism-corrected model performance. The apparent AUC was 0.744, and the optimism-corrected AUC/C-index was 0.725, with an AUC optimism of 0.019. The optimism-corrected calibration intercept was -0.159, the calibration slope was 0.918, and the Brier score was 0.072. These findings supported the acceptable internal performance of the final model, although external validation remains necessary. Detailed bootstrap validation results and the corresponding calibration curve are provided in **Supplementary Table S5** and **Supplementary Figure S2**, respectively.

**Figure 7:**
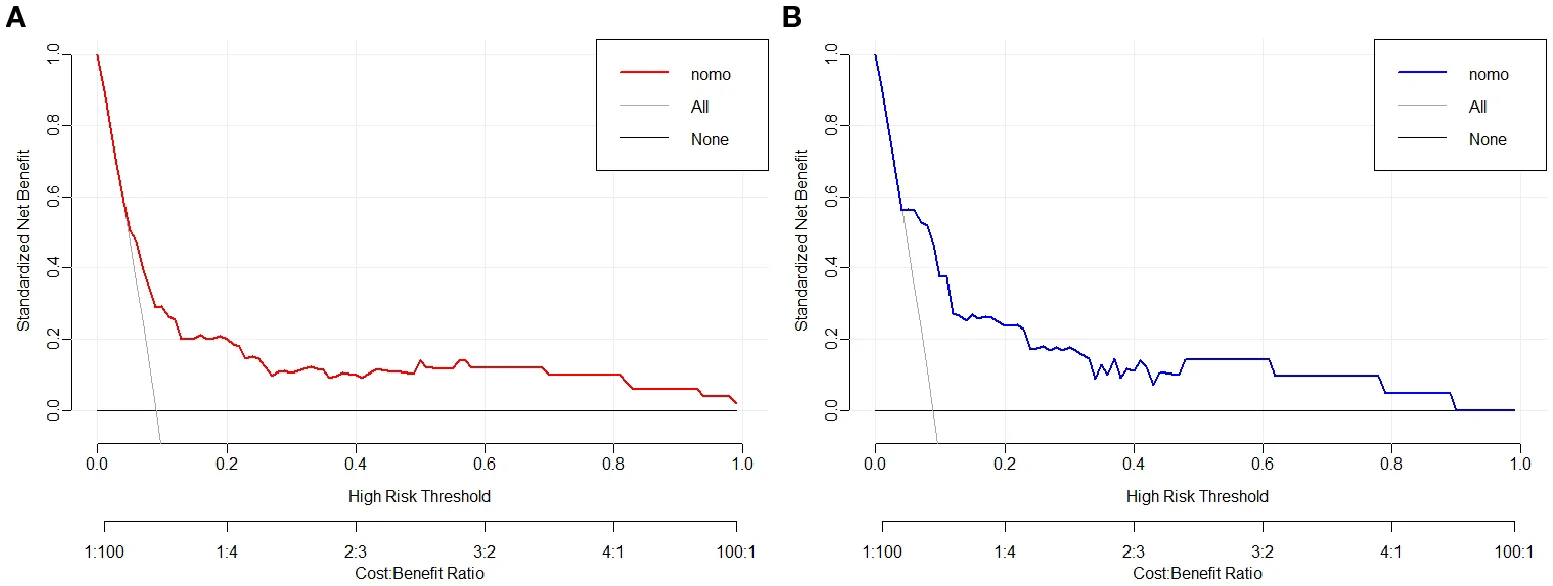
Decision curve analysis of the nomogram based on the training cohort **(A)** and validation cohort **(B)**.

Decision curve analysis (DCA) was used to evaluate the clinical utility of the model. The results showed that the nomogram yielded a higher net benefit across a threshold probability range of 0.02-0.99 in the training cohort and 0.03-0.91 in the validation cohort. These findings suggest that the model may provide a preliminary clinical reference for risk stratification and for identifying patients who may warrant further psychological screening within these threshold ranges (**Figure 7**).

## 4 Discussion

The main finding of this study is that clinically significant anxiety symptoms in patients with OSA were not fully captured by conventional respiratory severity indices alone. Instead, anxiety risk was associated with a combination of demographic, cardiovascular, inflammatory, hematological, anthropometric, and sleep-event-related characteristics. These findings should be interpreted as a multifactorial clinical risk profile rather than evidence of a distinct biological phenotype. A total of 787 patients with OSA were included in this study, and a nomogram was developed and internally validated to predict the probability of clinically significant anxiety symptoms. Using LASSO selection followed by post-LASSO multivariable logistic regression, six predictors were retained from 36 candidate variables: gender, cardiovascular disease, hemoglobin, neutrophil-to-lymphocyte ratio, BMI, and longest obstructive apnea duration. The present model should be interpreted as a prediction model rather than an explanatory or causal model. Predictors retained by LASSO may contribute to individualized risk estimation even when their individual regression coefficients do not reach statistical significance in the post-LASSO multivariable model. Therefore, the selected variables should be regarded as components of a risk prediction tool rather than established independent etiological determinants of anxiety symptoms. The model demonstrated acceptable discriminative ability, with AUCs of 0.735 and 0.751 in the training and validation cohorts, respectively. Calibration analysis also showed good agreement between predicted and observed risks, and the Hosmer-Lemeshow tests were non-significant in both the training and validation cohorts. Notably, anxiety symptoms were not simply associated with more severe conventional OSA indices. Compared with patients without anxiety symptoms, those with anxiety symptoms had relatively less severe OSA-related parameters, including lower AHI and ODI, shorter longest obstructive apnea duration, and higher minimum SpO_2_; however, they had poorer subjective sleep quality, as indicated by higher PSQI scores. These findings suggest that anxiety symptoms in patients with OSA may be associated with a multifactorial clinical risk profile that is not fully captured by conventional respiratory severity measures.

In addition, restricted cubic spline analysis demonstrated a significant overall association between longest obstructive apnea duration and anxiety symptoms: Wald χ² = 12.21, df = 3, P = 0.0067. Although the non-linear component showed only a borderline trend toward non-linearity, with a P value of 0.0583, categorical analysis showed that patients with a longest obstructive apnea duration of <60 seconds had a significantly higher risk of anxiety symptoms than those with a duration of 60–100 seconds: OR = 2.84, 95% CI 1.43-5.62, P = 0.0028. By contrast, the >100 seconds group showed a numerically higher but statistically non-significant risk: OR = 1.26, 95% CI 0.46-3.50, P = 0.654. These findings suggest that a shorter longest obstructive apnea duration may have potential value in anxiety risk assessment, whereas the observed non-linear pattern should be interpreted as exploratory.

Previous studies have increasingly recognized anxiety and depression as important psychological comorbidities in patients with OSA (11–18). The findings of the present study are broadly consistent with this body of literature, indicating that anxiety symptoms in patients with OSA are associated with specific demographic, clinical, laboratory, and sleep-related characteristics. However, our study further extends previous evidence by suggesting that anxiety risk may be predicted using a set of routinely available clinical, laboratory, and polysomnographic variables, rather than relying solely on AHI or oxygen desaturation metrics. In the present study, patients with anxiety symptoms had lower AHI and ODI, higher minimum SpO_2_, and shorter longest obstructive apnea duration. This finding is not entirely consistent with the traditional assumption that greater OSA severity necessarily corresponds to greater psychological burden. This discrepancy may be attributable to the discordance between objective respiratory severity and subjective sleep experience. Anxiety may be more closely related to sleep fragmentation, repeated arousals, heightened interoceptive sensitivity, insomnia symptoms, and perceived sleep insufficiency than to the absolute number of respiratory events alone (19–22). For example, Lee et al. reported that the relationship between OSA severity and depressive and anxiety symptoms was not simply linear, whereas subjective OSA symptoms showed a more consistent association with psychological symptoms (19). This observation is partly consistent with our finding that patients in the anxiety-symptom group had less severe conventional respiratory indices but poorer subjective sleep quality, as reflected by higher PSQI scores.

Importantly, although minimum SpO_2_ was included among the candidate PSG variables, the available data did not comprehensively characterize nocturnal hypoxemia. Mean SpO_2_, T90, and event-based hypoxic burden were not systematically available, and the absence of continuous raw SpO_2_ signals precluded reliable *post hoc* calculation of these metrics. These measures may capture the cumulative depth and duration of hypoxemia that are not fully represented by AHI, ODI, or a single oxygenation nadir (23). Emerging evidence suggests that hypoxic burden and average oxygen saturation may be associated with neuropsychiatric outcomes, particularly cognitive impairment (24, 25), whereas the relationship between OSA severity-related physiological indices and anxiety symptoms remains heterogeneous (19, 26). More direct measures of sleep fragmentation and arousal burden, including arousal index, respiratory arousal index, sleep efficiency, wake after sleep onset, and sleep-stage distribution, were also unavailable in the present retrospective dataset. Future prospective studies should jointly evaluate comprehensive oxygenation, arousal, and sleep-architecture metrics to determine their incremental predictive value for anxiety symptoms in OSA.

This discordance between subjective sleep quality and objective respiratory severity also highlights the need to consider the complex relationship between anxiety symptoms and sleep complaints. The relationship between anxiety symptoms and poor subjective sleep quality is likely bidirectional. Anxiety symptoms may worsen perceived sleep quality through cognitive hyperarousal, rumination, heightened interoceptive sensitivity, and increased awareness of nocturnal discomfort. Conversely, persistent poor sleep quality and insomnia-related symptoms may increase vulnerability to anxiety by impairing emotional regulation, increasing fatigue, and amplifying daytime functional impairment. This bidirectional relationship may partly explain why patients with SAS-defined anxiety symptoms had higher PSQI scores despite relatively milder PSG-derived respiratory indices. In addition, an insomnia-related OSA phenotype may contribute to the discordance between subjective sleep complaints and objective respiratory severity. Therefore, poorer PSQI scores in the anxiety-symptom group should be interpreted as reflecting a complex interaction among anxiety symptoms, subjective sleep experience, and insomnia-related complaints rather than as a direct consequence of respiratory severity alone. Because the present study was cross-sectional and did not include detailed insomnia-specific measures, the temporal direction of these associations could not be determined.

The association between shorter longest obstructive apnea duration and increased anxiety-symptom risk deserves careful interpretation. In the present study, patients with a longest obstructive apnea duration of <60 seconds had a higher risk of SAS-defined anxiety symptoms than those with a duration of 60–100 seconds (OR = 2.84, 95% CI 1.43-5.62, P = 0.0028). At first glance, this finding may appear counterintuitive, because longer respiratory events and greater hypoxemia are traditionally considered markers of increased OSA-related physiological stress. However, longest obstructive apnea duration represents the maximum duration of a single obstructive apnea event rather than the cumulative burden of nocturnal hypoxemia or total respiratory-event exposure. Therefore, it may capture a different dimension of sleep-disordered breathing from AHI, ODI, or minimum SpO_2_. One possible explanation is that shorter obstructive apnea duration may reflect a lower respiratory arousal threshold, meaning that patients may awaken rapidly in response to relatively brief respiratory disturbances. Repeated arousals may contribute to sleep fragmentation, sympathetic activation, cognitive and physiological hyperarousal, heightened perception of nocturnal discomfort, and poorer subjective sleep quality, all of which may be associated with anxiety symptoms (20–22). Conversely, longer respiratory events may indicate greater physiological tolerance to respiratory disturbance or a higher arousal threshold, although this interpretation remains speculative. This hypothesis may partly explain why patients in the anxiety-symptom group exhibited poorer subjective sleep quality, as indicated by higher PSQI scores, despite having relatively milder PSG-derived respiratory indices. Given the cross-sectional design of the present study and the non-significant association observed in the >100-second group, this finding should be regarded as exploratory and requires validation in future studies incorporating more detailed measures of arousal burden, sleep fragmentation, and hypoxic burden.

This study showed that gender was retained as a predictor of clinically significant anxiety symptoms in patients with OSA. Female patients with OSA may be less likely to present with classic symptoms such as loud snoring and witnessed apnea, and more likely to report insomnia, fatigue, mood disturbances, or anxiety-like complaints (27, 28). In the present study, although the overall cohort was predominantly male, the anxiety-symptom group had a higher proportion of female patients. This finding may reflect sex-related differences in symptom perception, neuroendocrine regulation, autonomic reactivity, and healthcare-seeking behavior. Therefore, our findings support the view that psychological screening should not be restricted to patients with severe polysomnographic abnormalities. Greater attention should be given to the assessment of psychological symptoms, particularly in female patients or those with prominent subjective sleep complaints.

Our study also indicated that cardiovascular disease was a predictor of anxiety symptoms in patients with OSA, further suggesting that anxiety in this population may be related to systemic disease burden. Cardiovascular disease may contribute to anxiety through several pathways, including increased health-related concerns, autonomic dysfunction, endothelial dysfunction, and chronic inflammatory activation (5–10). Conversely, anxiety may exacerbate cardiovascular risk through sympathetic overactivation, impaired sleep quality, reduced treatment adherence, and maladaptive behavioral responses. Although the present study could not determine the direction of causality, the retention of cardiovascular disease as a predictor in the model suggests that psychological risk assessment may be particularly important in OSA patients with comorbid cardiovascular disease.

The retention of NLR and hemoglobin in the model further supports a multidimensional interpretation of anxiety risk in patients with OSA. NLR is a readily available marker of systemic inflammation and inflammatory-immune status. Intermittent hypoxia and sleep fragmentation in OSA may promote oxidative stress and inflammatory activation, while inflammatory processes have also been implicated in anxiety-related pathophysiology (29–31). Although NLR was retained in the present model, other systemic inflammation- and immune-nutrition-based biomarkers were not evaluated. Composite indices such as the systemic immune-inflammation index, systemic inflammation response index, C-reactive protein-to-albumin ratio, platelet-to-lymphocyte ratio, monocyte-to-lymphocyte ratio, and prognostic nutritional index have been investigated in OSA and other respiratory diseases and may provide complementary information regarding systemic inflammatory and nutritional status. However, the present study was based on routinely extracted retrospective data, and not all component variables required for these indices were consistently available and quality-controlled in the full analytical cohort. Moreover, given the limited number of anxiety events, *post hoc* expansion of the candidate predictor set with multiple correlated inflammatory indices may increase model complexity and overfitting risk. Therefore, NLR should be interpreted as a readily available inflammatory-immune marker within a preliminary prediction model rather than as a comprehensive representation of systemic inflammation. Future prospective studies should evaluate whether multiple inflammatory, immune-nutritional, and dynamic biomarker trajectories can improve anxiety-risk prediction in patients with OSA. Hemoglobin may reflect hypoxia-related compensatory responses, oxygen-carrying capacity, or broader systemic physiological status. Recent systematic reviews and meta-analyses have suggested a potential association between OSA and hemoglobin levels, although this relationship may be influenced by disease severity, hypoxic burden, and population heterogeneity (31). Therefore, in the present study, Hb should be interpreted primarily as a component of the prediction model rather than as an established causal determinant.

BMI was the only anthropometric variable consistently available in the present cohort and was retained as a component of the prediction model. However, BMI provides limited information regarding regional fat distribution, central adiposity, and upper-airway soft-tissue burden. Other routinely obtainable measures, particularly neck circumference and waist circumference, may therefore offer complementary information. A recent study reported associations of neck and waist circumference with PSG-defined OSA severity, although the correlations with AHI were modest and BMI showed limited association (32). Because neck and waist circumference were not systematically recorded and quality-controlled for the full cohort, they could not be reliably incorporated into the current model. Moreover, evidence linking these measures to OSA severity does not necessarily establish their predictive value for anxiety symptoms. Future prospective studies should evaluate whether comprehensive anthropometric assessment, including BMI, neck circumference, waist circumference, waist-to-hip ratio, and relevant upper-airway anatomical measures, provides incremental value for psychological-risk prediction in OSA.

In addition to these physiological interpretations, several alternative explanations should be considered. First, referral bias may have influenced the observed associations because the study population consisted of hospitalized patients from a single otolaryngology department. These patients may differ from community-based OSA populations or patients recruited from general sleep-medicine clinics in terms of symptom burden, referral pathways, treatment expectations, and comorbidity profiles. Second, symptom-reporting differences may partly explain why patients with anxiety symptoms had poorer subjective sleep quality despite relatively milder PSG-derived respiratory indices. Patients with anxiety symptoms may be more likely to perceive, recall, and report sleep-related discomfort, nocturnal symptoms, or daytime impairment. Third, an insomnia-related OSA phenotype may have contributed to the observed pattern, because insomnia complaints and anxiety symptoms frequently coexist and may be more strongly reflected by subjective sleep quality measures than by AHI or ODI alone. Fourth, sex-related differences in symptom perception and healthcare-seeking behavior may have contributed to the higher proportion of female patients in the anxiety-symptom group. Finally, residual confounding from unmeasured factors, such as detailed insomnia severity, prior psychiatric history, socioeconomic status, medication use, psychosocial stressors, and healthcare-seeking behavior, cannot be excluded. Therefore, the observed associations should be interpreted cautiously and should not be regarded as evidence of causal physiological pathways.

From a clinical perspective, once routinely available clinical, laboratory, and PSG data have been collected, the nomogram may serve as a preliminary triage aid for estimating the probability of SAS-defined anxiety symptoms in patients with OSA (33–40). Its potential value lies in identifying patients who may warrant standardized psychological assessment, including those whose psychological burden might otherwise be overlooked because conventional PSG severity indices such as AHI and ODI are not markedly elevated. A higher model-estimated probability should prompt further standardized anxiety assessment and a focused clinical interview rather than directly establish an anxiety diagnosis. Psychological or psychiatric referral may be considered when subsequent assessment identifies severe or persistent symptoms, clinically meaningful functional impairment, marked emotional distress, diagnostic uncertainty, or safety concerns. However, the present study did not establish an externally validated probability threshold for screening or referral. Therefore, the nomogram should not be used as a stand-alone referral rule or as a substitute for clinical judgment, validated psychological assessment, or formal psychiatric evaluation. External validation and clinical-impact studies are required before broader implementation and before specific risk thresholds can be recommended.

## 5 Limitations

Several limitations should be acknowledged. First, the observational design of this study precludes causal inference between the model-selected predictors and anxiety symptoms. Therefore, the variables retained in the model should be interpreted as predictors rather than established etiological determinants.

Second, the relatively small number of anxiety events is an important methodological limitation. Although 787 patients were included in the final analysis, only 71 patients had clinically significant anxiety symptoms. Given that 36 candidate predictors were initially considered during model development, the limited number of events may have affected model stability and increased the risk of overfitting. LASSO logistic regression was used to reduce model complexity through penalization and variable shrinkage, and only six predictors with non-zero coefficients were retained for the post-LASSO multivariable model. Nevertheless, penalized regression cannot fully eliminate the uncertainty associated with a low event-to-variable ratio. Therefore, the present model should be interpreted as an internally validated risk stratification tool rather than a definitive prediction model.

Third, internal validation was primarily performed using a random 7:3 split into training and validation cohorts. Although this approach provided a validation subset independent of model fitting, it may have reduced statistical efficiency given the limited number of outcome events. We therefore additionally performed bootstrap-based internal validation using the entire analytical cohort with 1,000 resamples. Nevertheless, both approaches represent internal validation and cannot establish the model’s transportability to independent populations or different clinical settings. The absence of external validation is therefore a major limitation of the present study. Future multicenter prospective studies should evaluate the model’s discrimination, calibration, and clinical utility in independent cohorts from different geographic regions, sleep laboratories, and clinical settings, and should consider model updating if performance varies across populations.

Fourth, several potential sources of bias should be considered. Selection bias may have been introduced because all participants were recruited from a single-center hospitalized otolaryngology population. Compared with the broader OSA population, patients evaluated in an otolaryngology department may have more prominent anatomical upper-airway abnormalities, stronger indications for surgical or upper-airway assessment, different referral pathways, greater symptom burden, different treatment expectations, and distinct comorbidity profiles. Therefore, the present cohort may not be representative of community-based OSA patients, primary-care populations, psychiatric settings, or general sleep-medicine clinics. Residual confounding also cannot be excluded. Although we collected demographic characteristics, comorbidities, laboratory parameters, patient-reported scales, and PSG variables, some potentially relevant factors were unavailable or incompletely measured, including neck circumference, waist circumference, upper-airway anatomical measurements, detailed insomnia severity, prior or mild psychiatric history, socioeconomic status, psychosocial stressors, medication use, healthcare-seeking behavior, and depressive symptoms. Anxiety and depression frequently coexist in patients with OSA, and depressive symptoms may influence subjective sleep quality, daytime functioning, symptom reporting, and overall psychological distress. Because depression was not systematically incorporated into the present prediction model, we could not determine whether the identified predictors were specifically associated with anxiety symptoms or reflected a broader psychological distress profile involving both anxiety and depressive symptoms. Future prospective studies should assess anxiety and depression simultaneously, preferably using validated symptom scales and structured psychiatric interviews.

Fifth, misclassification bias should be acknowledged because anxiety symptoms were defined using the SAS rather than a structured psychiatric diagnostic interview. Although the SAS is feasible and commonly used as a self-report screening instrument in clinical settings, it cannot establish a formal diagnosis of anxiety disorder. Therefore, the outcome of this study should be interpreted as SAS-defined clinically significant anxiety symptoms rather than clinically diagnosed anxiety disorder. Moreover, other validated anxiety screening tools, such as the Generalized Anxiety Disorder-7 scale (GAD-7), were not routinely collected in this retrospective dataset. Future prospective studies should incorporate structured psychiatric interviews or multiple validated anxiety screening instruments to improve outcome classification.

Sixth, the study population was predominantly male, with men accounting for 89.3% of the cohort. This sex imbalance may limit the generalizability of the findings, particularly because sex itself was retained as an important predictor and OSA-related anxiety symptoms may differ between men and women.

Seventh, the retrospective PSG dataset lacked several potentially informative measures of nocturnal hypoxemia, sleep fragmentation, and arousal burden. Although AHI, ODI, minimum SpO_2_, and longest obstructive apnea duration were available, mean SpO_2_, T90, event-based hypoxic burden, arousal index, respiratory arousal index, sleep efficiency, wake after sleep onset, and sleep-stage distribution were not systematically available for the full cohort. In particular, the absence of continuous raw SpO_2_ signals prevented reliable *post hoc* calculation of event-based hypoxic burden. This may have limited both the physiological characterization of OSA and the predictive completeness of the model. Future prospective studies should preserve raw PSG signals and evaluate comprehensive oxygenation, arousal, sleep-fragmentation, and sleep-architecture metrics jointly.

Eighth, the non-linear association between longest obstructive apnea duration and anxiety symptoms should be regarded as an exploratory finding. Although the overall association was statistically significant (Wald χ² = 12.21, P = 0.0067), the non-linear component only approached statistical significance (P = 0.0583). In addition, the association between the >100-second group and anxiety risk was not statistically significant (OR = 1.26, 95% CI 0.46-3.50, P = 0.654). Larger studies are needed to determine whether this asymmetric U-shaped pattern is reproducible.

Finally, several potentially informative systemic inflammation- and immune-nutrition-based biomarkers, including SII/SIII, SIRI, CAR, PLR, MLR, and PNI, were not evaluated because the required component variables were not consistently available and quality-controlled for the full retrospective cohort. Future prospective studies should incorporate these indices and assess whether they provide incremental predictive value beyond NLR.

## 6 Conclusions

In conclusion, this study developed and internally validated a LASSO-based nomogram for predicting SAS-defined clinically significant anxiety symptoms in patients with obstructive sleep apnea. The model incorporated gender, cardiovascular disease, hemoglobin, NLR, BMI, and longest obstructive apnea duration, and demonstrated acceptable internal performance. These findings suggest that anxiety-symptom risk in obstructive sleep apnea may not be fully captured by conventional respiratory severity indices alone, but may instead be associated with a multifactorial clinical risk profile. However, given the single-center retrospective design, limited number of anxiety events, questionnaire-based outcome definition, and lack of external validation, this nomogram should be regarded as a preliminary risk stratification tool rather than a model ready for immediate clinical implementation. Future multicenter prospective studies are needed to externally validate the model and to determine whether model-assisted psychological screening can improve clinical assessment and patient management.

## Data Availability

The original contributions presented in the study are included in the article/**Supplementary Material**. Further inquiries can be directed to the corresponding author.
