# Supplementary material for: Development and internal validation of a LASSO-based nomogram for predicting SAS-defined anxiety symptoms in obstructive sleep apnea
Source: Front Psychiatry. 2026 Sep 8;17:1899687. doi: 10.3389/fpsyt.2026.1899687 (PMC13593852; doi:10.3389/fpsyt.2026.1899687)
Supplement: Supplementary file 1 [file SupplementaryFile1.docx]

**Supplementary Methods**

**Restricted cubic spline and categorical sensitivity analyses**

Restricted cubic spline (RCS) analysis was conducted to examine the potential non-linear association between longest obstructive apnea duration and clinically significant anxiety symptoms, defined as an SAS standardized score ≥50. The RCS model was fitted using the rms package in R. Four knots were placed at the 5th, 35th, 65th, and 95th percentiles of longest obstructive apnea duration, corresponding to 41.0, 68.3, 76.0, and 113.1 seconds, respectively. The reference value was 72.6 seconds. The model was adjusted for apnea–hypopnea index (AHI), oxygen desaturation index (ODI), age, and body mass index (BMI).

To complement the RCS analysis and evaluate the robustness of the observed pattern, longest obstructive apnea duration was further categorized into three groups: <60 s, 60-100 s, and >100 s. The 60-100 s group was selected as the reference because the RCS curve suggested the lowest estimated risk at approximately 91.73 s, which fell within this interval. Adjusted odds ratios (ORs), 95% confidence intervals (CIs), and P values were estimated using logistic regression adjusted for AHI, ODI, age, and BMI.

**Supplementary Table S1. Baseline characteristics of study population**

| **Variables** | **Total** | **Training** | **Validation** | **p value** | **t/(Z)/[χ²] value** |
| --- | --- | --- | --- | --- | --- |
| **N** | 787 | 551 | 236 |  |  |
| **Demographic and clinical characteristics:** | | | | | |
| Disease duration (year) | 10.00  (4.00, 10.00) | 10.00  (4.00, 10.00) | 8.00  (3.00, 10.00) | 0.138 | (-1.483) |
| Age (year) | 40.00  (36.00, 46.00) | 40.00  (36.00, 46.00) | 40.00  (35.00, 45.25) | 0.482 | (-0.702) |
| Gender/ n (%) | | | | | |
| Female | 84 (10.7) | 52 (9.4) | 32 (13.6) | 0.112 | [2.944] |
| Male | 703 (89.3) | 499 (90.6) | 204 (86.4) |  |  |
| Smoking History/ n (%) | | | | | |
| No | 517 (65.7) | 358 (65.0) | 159 (67.4) | 0.570 | [0.422] |
| Yes | 270 (34.3) | 193 (35.0) | 77 (32.6) |  |  |
| Alcohol Consumption History/ n (%) | | | | | |
| No | 474 (60.2) | 335 (60.8) | 139 (58.9) | 0.675 | [0.249] |
| Yes | 313 (39.8) | 216 (39.2) | 97 (41.1) |  |  |
| BMI (kg/m^2^) | 28.37  (25.79, 31.14) | 28.40  (25.95, 31.14) | 28.11  (25.54, 31.14) | 0.523 | (-0.639) |
| Hypertension/ n (%) | | | | | |
| No | 518 (65.8) | 362 (65.7) | 156 (66.1) | 0.978 | [0.012] |
| Yes | 269 (34.2) | 189 (34.3) | 80 (33.9) |  |  |
| DM/ n (%) | | | | | |
| No | 729 (92.6) | 508 (92.2) | 221 (93.6) | 0.573 | [0.508] |
| Yes | 58 (7.4) | 43 (7.8) | 15 (6.4) |  |  |
| Cardiovascular Disease/ n (%) | | | | | |
| No | 677 (86.0) | 467 (84.8) | 210 (89.0) | 0.146 | [2.457] |
| Yes | 110 (14.0) | 84 (15.2) | 26 (11.0) |  |  |
| Inferior Turbinate Hypertrophy/ n (%) | | | | | |
| No | 44 (5.6) | 25 (4.5) | 19 (8.1) | 0.072 | [3.865] |
| Yes | 743 (94.4) | 526 (95.5) | 217 (91.9) |  |  |
| Fatty Liver Disease/ n (%) | | | | | |
| No | 518 (65.8) | 355 (64.4) | 163 (69.1) | 0.240 | [1.581] |
| Yes | 269 (34.2) | 196 (35.6) | 73 (30.9) |  |  |
| Abnormal Pulmonary Imaging Findings/ n (%) | | | | | |
| No | 100 (12.7) | 65 (11.8) | 35 (14.8) | 0.292 | [1.371] |
| Yes | 687 (87.3) | 486 (88.2) | 201 (85.2) |  |  |
| Chronic Rhinitis/ n (%) | | | | | |
| No | 645 (82.0) | 452 (82.0) | 193 (81.8) | 1.000 | [0.007] |
| Yes | 142 (18.0) | 99 (18.0) | 43 (18.2) |  |  |
| Hyperlipidemia/ n (%) | | | | | |
| No | 514 (65.3) | 367 (66.6) | 147 (62.3) | 0.278 | [1.360] |
| Yes | 273 (34.7) | 184 (33.4) | 89 (37.7) |  |  |
| Memory Impairment/ n (%) | | | | | |
| No | 163 (20.7) | 83 (15.1) | 35 (14.8) | 0.110 | [7.099] |
| Yes | 624 (79.3) | 468 (84.9) | 201 (85.2) |  |  |
| **OSA-related parameters:** | | | | | |
| AHI (events/h) | 57.90  (41.10, 69.60) | 57.90  (41.95, 69.95) | 56.65  (37.70, 69.15) | 0.188 | (-1.316) |
| Longest Obstructive Apnea Duration (s) | 72.60  (61.60, 85.75) | 72.60  (61.80, 84.00) | 72.60  (60.90, 91.12) | 0.445 | (-0.764) |
| ODI (events/h) | 54.40  (39.40, 65.50) | 54.40  (41.55, 66.95) | 54.40  (33.40, 64.10) | 0.336 | (-0.962) |
| minSpO_2_ (%) | 67.00  (55.00, 78.00) | 67.00  (55.00, 77.50) | 67.00  (54.00, 79.00) | 0.570 | (-0.568) |
| **Patient-reported outcomes:** | | | | | |
| PSQI | 8.00 (6.00, 10.00) | 8.00 (7.00, 10.00) | 8.00 (6.00, 10.00) | 0.364 | (-0.908) |
| ESS | 7.00 (4.00, 11.00) | 7.00 (4.00, 11.00) | 7.00 (4.00, 11.00) | 0.514 | (-0.652) |
| **Laboratory parameters** | | | | | |
| Urinary Creatinine (mmol/L) | 17.70  (8.80, 26.50) | 17.70 (8.80, 26.50) | 17.70  (8.80, 26.50) | 0.743 | (-0.328) |
| HDL-C (mmol/L) | 1.13 (0.97, 1.30) | 1.12 (0.97, 1.29) | 1.15 (0.99, 1.32) | 0.479 | (-0707) |
| FBG (mmol/L) | 5.15 (4.77, 5.64) | 5.14 (4.76, 5.62) | 5.18 (4.81, 5.73) | 0.573 | (-0.563) |
| TG (mmol/L) | 1.94 (1.33, 2.88) | 1.95 (1.34, 2.92) | 1.88 (1.28, 2.73) | 0.192 | (-1.304) |
| FT4 (pmol/L) | 11.15  (10.22, 12.17) | 11.20  (10.24, 12.27) | 11.07  (10.20, 11.92) | 0.206 | (-1.265) |
| FT3 (pmol/L) | 5.54 (5.13, 5.99) | 5.53 (5.13, 5.97) | 5.55 (5.14, 6.02) | 0.674 | (-0.420) |
| TC (mmol/L) | 5.05 (4.46, 5.85) | 5.03 (4.46, 5.86) | 5.11 (4.47, 5.85) | 0.658 | (-0.442) |
| UA (μmol/L) | 402.40 ± 92.97 | 403.55 ± 89.54 | 399.69 ± 100.69 | 0.594 | 0.533 |
| TSH (mIU/L) | 1.72 (1.25, 2.40) | 1.71 (1.25, 2.37) | 1.77 (1.25, 2.47) | 0.593 | (-0.534) |
| LDL-C (mmol/L) | 3.09 (2.64, 3.58) | 3.08 (2.62, 3.58) | 3.12 (2.66, 3.61) | 0.639 | (-0.469) |
| NLR | 1.88 (1.49, 2.48) | 1.87 (1.48, 2.41) | 1.89 (1.49, 2.64) | 0.144 | (-1.460) |
| Hb (g/L) | 155.00  (146.00, 163.00) | 155.00  (146.00, 163.00) | 153.00  (145.00, 163.00) | 0.424 | (-0.799) |
| Platelet Count (×10^9^/L) | 234.00  (202.00, 274.00) | 235.00  (202.50, 273.00) | 233.00  (202.00, 276.00) | 0.624 | (-0.490) |
| WBC (×10^9^/L) | 6.50 (5.45, 7.70) | 6.60 (5.50, 7.75) | 6.30 (5.30, 7.50) | 0.080 | (-1.748) |
| eGFR (mL/min/1.73m²) | 116.14  (107.40, 123.84) | 115.96  (107.36, 123.78) | 116.67  (107.62, 123.90) | 0.504 | (-0.668) |

**Notes:** Data are presented as mean ± standard deviation, median (interquartile range), or number (percentage), as appropriate. P values were calculated using Student’s t test or Mann-Whitney U test for continuous variables and χ² test or Fisher’s exact test for categorical variables.

**Abbreviations:** AHI, Apnea-Hypopnea Index; ODI, Oxygen Desaturation Index; PSQI, Pittsburgh Sleep Quality Index; ESS, Epworth Sleepiness Scale; HDL-C, high-density lipoprotein cholesterol; LDL-C, low-density lipoprotein cholesterol; eGFR, estimated glomerular filtration rate; NLR, neutrophil-to-lymphocyte ratio; WBC, white blood cell count.

**Supplementary Table S2. Association between categorized longest obstructive apnea duration and clinically significant anxiety symptoms**

| **Longest obstructive apnea duration** | **n** | **OR value** | **95% CI** | **p value** |
| --- | --- | --- | --- | --- |
| 60-100 s | 372 | Reference | Reference | - |
| <60 s | 121 | 2.84 | 1.43-5.62 | 0.0028 |
| >100 s | 58 | 1.26 | 0.46-3.50 | 0.654 |

**Note:** Odds ratios were adjusted for AHI, ODI, age, and BMI. Clinically significant anxiety symptoms were defined as an SAS standardized score ≥50. The 60-100 s group was used as the reference group.

**Supplementary Table S3. Comparison of linear and restricted cubic spline models**

| **Model** | **LogLik** | **Parameters** | **AIC** | **BIC** |
| --- | --- | --- | --- | --- |
| Linear | −157.62 | 6 | 327.25 | 353.12 |
| Restricted cubic spline | −155.14 | 8 | 326.27 | 360.77 |

**Likelihood ratio test:** χ²=4.9717, df=2, *P*=0.0833**.**

**Note:** The restricted cubic spline model showed a slightly lower AIC than the linear model, but a higher BIC. The likelihood ratio test did not reach statistical significance, suggesting that the spline model may capture a possible non-linear pattern, although evidence for non-linearity remained exploratory.

**Supplementary Figure S1. Restricted cubic spline analysis of the association between longest obstructive apnea duration and clinically significant anxiety symptoms.**

The restricted cubic spline model was fitted with four knots located at the 5th, 35th, 65th, and 95th percentiles of longest obstructive apnea duration, corresponding to 41.0, 68.3, 76.0, and 113.1 seconds, respectively. The model was adjusted for AHI, ODI, age, and BMI. Odds ratios were plotted relative to the reference value of 72.6 seconds. The estimated nadir of predicted risk was approximately 91.73 seconds. The overall association was statistically significant (P=0.0067), whereas the non-linear component showed a borderline trend (P for non-linearity=0.0583).

**Supplementary Results.**

**Non-linear and categorical analyses of longest obstructive apnea duration**

In the RCS analysis, longest obstructive apnea duration was significantly associated with clinically significant anxiety symptoms overall (Wald χ²=12.21, df=3, P=0.0067), while the non-linear component showed a borderline trend (χ²=5.68, df=2, P=0.0583). The estimated nadir of predicted risk was observed at approximately 91.73 seconds.

The linear and spline models were further compared. The restricted cubic spline model had a slightly lower AIC than the linear model (326.27 vs 327.25), but a higher BIC (360.77 vs 353.12). The likelihood ratio test comparing the spline and linear models did not reach statistical significance (χ²=4.97, df=2, P=0.0833), indicating that evidence for improved fit with the spline specification was suggestive but not definitive.

In categorical analysis, patients with longest obstructive apnea duration <60 s had significantly higher odds of clinically significant anxiety symptoms compared with those in the 60-100 s reference group (OR=2.84, 95% CI 1.43-5.62, P=0.0028). The >100 s group showed a non-significant increase in risk (OR=1.26, 95% CI 0.46-3.50, P=0.654). These findings support the robustness of the observation that shorter obstructive apnea duration may be associated with increased anxiety symptom risk, while the overall non-linear association should be interpreted as exploratory.

**Supplementary Table S4. Missing data profile of candidate predictors and imputation methods**

| **Variable** | **Training / Missing** | | **Validation/Missing** | | **Imputation**  **method** | **Handling** |
| --- | --- | --- | --- | --- | --- | --- |
|  | **n** | **percent** | **n** | **percent** |  |  |
| Urinary  Creatinine | 39 | 7.08 | 14 | 5.93 | Median | Median imputation using training-cohort value |
| FBG | 3 | 0.54 | 1 | 0.42 | Median | Median imputation using training-cohort value |
| FT3 | 3 | 0.54 | 2 | 0.85 | Median | Median imputation using training-cohort value |
| FT4 | 3 | 0.54 | 2 | 0.85 | Median | Median imputation using training-cohort value |
| TSH | 3 | 0.54 | 2 | 0.85 | Median | Median imputation using training-cohort value |
| HDL-C | 2 | 0.36 | 2 | 0.85 | Median | Median imputation using training-cohort value |
| LDL-C | 2 | 0.36 | 2 | 0.85 | Median | Median imputation using training-cohort value |
| Longest  Obstructive Apnea Duration | 2 | 0.36 | 0 | 0 | Median | Median imputation using training-cohort value |
| ODI | 2 | 0.36 | 0 | 0 | Median | Median imputation using training-cohort value |
| TC | 2 | 0.36 | 2 | 0.85 | Median | Median imputation using training-cohort value |
| TG | 2 | 0.36 | 2 | 0.85 | Median | Median imputation using training-cohort value |
| minSpO_2_. | 2 | 0.36 | 0 | 0 | Median | Median imputation using training-cohort value |
| AHI | 1 | 0.18 | 0 | 0 | Median | Median imputation using training-cohort value |
| Hb | 1 | 0.18 | 0 | 0 | Median | Median imputation using training-cohort value |
| NLR | 1 | 0.18 | 0 | 0 | Median | Median imputation using training-cohort value |
| Platelet Count | 1 | 0.18 | 0 | 0 | Median | Median imputation using training-cohort value |
| WBC | 1 | 0.18 | 0 | 0 | Median | Median imputation using training-cohort value |
| Abnormal Pulmonary Imaging Findings | 0 | 0 | 0 | 0 | Mode | Mode imputation using training-cohort value |
| Age | 0 | 0 | 0 | 0 | Median | Median imputation using training-cohort value |
| Alcohol Consumption History | 0 | 0 | 0 | 0 | Mode | Mode imputation using training-cohort value |
| BMI | 0 | 0 | 0 | 0 | Median | Median imputation using training-cohort value |
| Cardiovascular Disease | 0 | 0 | 0 | 0 | Mode | Mode imputation using training-cohort value |
| Chronic Rhinitis | 0 | 0 | 0 | 0 | Mode | Mode imputation using training-cohort value |
| DM | 0 | 0 | 0 | 0 | Mode | Mode imputation using training-cohort value |
| Diseaseduration | 0 | 0 | 0 | 0 | Median | Median imputation using training-cohort value |
| ESS | 0 | 0 | 0 | 0 | Median | Median imputation using training-cohort value |
| Egfr | 0 | 0 | 2 | 0.85 | Median | Median imputation using training-cohort value |
| FattyLiver Disease | 0 | 0 | 0 | 0 | Mode | Mode imputation using training-cohort value |
| Gender | 0 | 0 | 0 | 0 | Mode | Mode imputation using training-cohort value |
| Hyperlipidemia | 0 | 0 | 0 | 0 | Mode | Mode imputation using training-cohort value |
| Hypertension | 0 | 0 | 0 | 0 | Mode | Mode imputation using training-cohort value |
| Inferior Turbinate Hypertrophy | 0 | 0 | 0 | 0 | Mode | Mode imputation using training-cohort value |
| Memory Impairment | 0 | 0 | 0 | 0 | Mode | Mode imputation using training-cohort value |
| PSQI | 0 | 0 | 0 | 0 | Median | Median imputation using training-cohort value |
| Smoking History | 0 | 0 | 0 | 0 | Mode | Mode imputation using training-cohort value |
| UA | 0 | 0 | 2 | 0.85 | Median | Median imputation using training-cohort value |

**Supplementary Table S5. Bootstrap-based internal validation of the final prediction model using the entire analytical cohort**

| **Metric** | **Value** |
| --- | --- |
| Apparent AUC | 0.7437 |
| Optimism-corrected AUC/C-index | 0.7247 |
| AUC optimism | 0.0191 |
| Calibration intercept | -0.1589 |
| Calibration slope | 0.9176 |
| Brier score | 0.0725 |

**Supplementary Figure S2. Bootstrap calibration curve of the final prediction model using the entire analytical cohort with 1,000 resamples.**

**Supplementary Table S6. TRIPOD reporting checklist.**

| **TRIPOD item** | **Reporting location in revised manuscript** |
| --- | --- |
| Study design | Methods 2.1 |
| Source population and inclusion pathway | Methods 2.1 |
| Eligibility criteria | Methods 2.1 |
| Outcome definition | Methods 2.3 |
| Candidate predictors | Methods 2.2 / 2.4 |
| Missing data handling | Methods 2.4; Supplementary Table S4 |
| Model development | Methods 2.4 |
| Internal validation | Methods 2.4; Results |
| Model performance | Results; Figures 5-7; Supplementary Table S5 |
| Model interpretation and limitations | Discussion |
| Generalizability | Discussion / Limitations |
